# Monkeypox Virus Infections in Southern Italy: Is There a Risk for Community Spread?

**DOI:** 10.3390/ijerph191811719

**Published:** 2022-09-17

**Authors:** Daniela Loconsole, Anna Sallustio, Francesca Centrone, Daniele Casulli, Marisa Accogli, Annalisa Saracino, Caterina Foti, Mauro Grandolfo, Giovanni Battista Buccoliero, Viviana Vitale, Sara De Nitto, Michele Conversano, Francesco Desiante, Laura Del Sambro, Domenico Simone, Antonio Parisi, Rosa Prato, Domenico Martinelli, Maria Chironna

**Affiliations:** 1 Hygiene Section, Department of Interdisciplinary Medicine, University of Bari, 70124 Bari, Italy; 2 Hygiene Unit, Azienda Ospedaliero-Universitaria Consorziale Policlinico di Bari, 70124 Bari, Italy; 3 Infectious Diseases Section, Department of Biomedical Sciences and Human Oncology, University of Bari, 70124 Bari, Italy; 4 Section of Dermatology, Department of Biomedical Sciences and Human Oncology, University of Bari, 70124 Bari, Italy; 5 Infectious Diseases Unit, San Giuseppe Moscati Hospital, 74010 Taranto, Italy; 6 Department of Prevention, Local Health Authority of Bari, 70100 Bari, Italy; 7 Department of Prevention, Local Health Authority of Taranto, 74121 Taranto, Italy; 8 Istituto Zooprofilattico Sperimentale della Puglia e della Basilicata, 71121 Foggia, Italy; 9 Hygiene Section, Medical and Surgical Sciences, University of Foggia, 71122 Foggia, Italy

**Keywords:** monkeypox virus, surveillance, epidemiology, whole-genome sequencing, southern Italy

## Abstract

The ongoing outbreak of the Monkeypox virus (MPXV) is characterized by sustained human-to-human transmission, particularly among men who have sex with men (MSM). The aim of the study was to describe the characteristics of the MPXV infection identified in Southern Italy. Clinical samples for each suspected case identified from 1 June to 1 August 2022 were tested for MPXV, and whole-genome sequencing (WGS) was performed on two strains. Ten cases were identified: eight were young adult males, including six MSMs, and two were female. Nine subjects reported recent sexual exposure. One female subject without sexual exposure only reported attendance at a social gathering. Overall, 7 of 10 skin lesion samples had a high viral load of MPXV DNA, and 6/9 whole blood samples and 6/8 nasopharyngeal swab samples also tested positive. The analyzed sequences belonged to Clade 3, lineage B.1, and B.1.5, respectively. Despite this recent multinational outbreak of MPXV cases having revealed a high proportion of cases occurring among MSM, the identification of cases among heterosexual subjects and in a female subject without sexual risk factors should raise awareness among clinicians about the possible spread of MPXV in the general population.

## 1. Introduction

A worldwide outbreak of Monkeypox virus (MPXV) infection in humans has been ongoing in non-endemic countries since May 2022. The emergence of this outbreak is regarded as a global threat, and the World Health Organization declared a Public Health Emergency of International Concern on 23 July 2022 [1]. Since the elimination of the smallpox virus in 1980, MPXV has been considered the most severe orthopoxvirus circulating in humans as an incidental host [2]. As of 27 July 2022, 20,638 cases have been identified in 77 countries worldwide, 71 of which had never reported an MPXV infection before this outbreak [3]. A total of 13,043 cases have been reported in the European region, with several cases reported among travelers returning from endemic countries [4,5,6]. 

After initially being recognized as a zoonotic pathogen, MPXV has frequently jumped from its reservoir hosts into humans—mainly in the Democratic Republic of Congo—whereas human-to-human transmission was low [7]. Previous outbreaks reported in African countries and the USA were caused mainly by spillover events from animals to humans [8,9]. 

Viruses can be transmitted through close contact with lesions and respiratory droplets and via fomites, such as bedding and towels [10]. The current outbreak is characterized by sustained human-to-human transmission among adult males, particularly among men who have sex with men (MSM) [10,11]. The predominance of cases among MSM and the nature of the presenting lesions suggest that transmission occurs during sexual intercourse [10]. Sexual transmission through skin-to-skin contact during sexual intercourse or via genital secretions was first hypothesized to occur during an MPXV outbreak in 2017 in Nigeria [12].

Clinical features of MPXV infections are similar to those caused by smallpox but with lower mortality and complication rates [13]. Many infected persons experience a mild, self-limiting course of the disease despite the absence of specific treatments [14]. In certain persons, however, the disease can be more severe, especially among young children, pregnant women, and immunocompromised subjects [15]. Following mild prodromal symptoms, such as fever, sore throat, and lymphadenopathy, patients infected with MPXV experience a rash with an appearance typical of pimples or blisters on the face, inside the mouth, and on other parts of the body [16]. Patients with disseminated rashes experience lesions on the palms and soles of the feet. The progression of the lesions is usually asynchronous [17]. Most patients infected during the current outbreak first experienced a rash in the genital and perianal areas, with or without lesions in other parts of the body [16]. This atypical presentation suggested that the genital area was the site of primary infection [7]. Currently, the diagnosis of MPXV infection is performed via MPXV real-time polymerase chain reactions (Real Time-PCR) on suspected skin lesions [10]. Due to the limited duration of viremia, scabs, swabs and aspirated lesion fluid should preferably be used for PCR over blood [10].

Historically, MPXV has been classified into two main clades, the Central African and West African clades [18]. More recently, MPXV was classified into three clades, MPXV Clade 1 (Congo Basin clade), Clade 2 (West African clade), and Clade 3, with the latter, including isolates identified during the most recent outbreaks [19]. The MPXV involved in the 2022 outbreak belongs to Clade 3, lineage B.1 [20]. Although this double-stranded DNA virus shows a low mutation frequency, a comparison with the strains isolated in 2018 has revealed 46 common mutations among the strains involved in the 2022 outbreak [2,21]. An in-depth mutational analysis also suggested a potential MPXV human adaptation during the ongoing microevolution [20].

Following the global alert of the spread of MPXV in non-endemic areas, the Italian Ministry of Health recommended the notification of suspected cases, contact tracing, isolation, and the appropriate management of cases. As of 27 July 2022, 426 cases have been identified in Italy—almost all in young males, including 129 (30.3%) acquired abroad [22]. The first four cases were reported between 17 and 22 May 2022 [17]. All patients were young MSM adults, two of whom were HIV positive, and reported traveling abroad during the weeks before the onset of symptoms [17]. Based on current data, the risk of a community spread of this virus could increase. The aim of the present study was to describe the epidemiological, clinical, and virological characteristics of the MPXV infection identified in the Apulia region of southern Italy from 1 June to 1 August 2022.

## 2. Materials and Methods

Because the activities described in this study were part of the legislated mandate of the Health Promotion and Public Health Department of the Apulia region (Italy), approval of this study by the Ethics Committee was waived. All procedures were performed in accordance with the Declaration of Helsinki, as revised in 2013, for research involving human subjects.

An integrated epidemiological and virological surveillance system for MPXV infection in Italy has been set up at the national level. According to this system, all suspected cases must be promptly reported to the local health authority within 12 h and subjected to laboratory testing according to the recommendations of the Ministry of Health [23]. In the Apulia region, surveillance activities were jointly performed by the teams for infectious diseases from the Regional Department of Health and the Laboratory of Molecular Epidemiology and Public Health of the Hygiene Unit (A.O.U.C. Policlinico Bari), which is the regional reference center for MPXV diagnosis. If a case was suspected, a form containing personal and clinical data, risk factors, and travel history was filled in. According to regional recommendations, skin lesion swabs, nasopharyngeal swabs, whole blood, and serum samples were collected for each case. Clinical cases were classified according to the Italian Ministry of Health classification system [23]. A case was defined as suspected if an individual had skin lesions and general symptoms—as probable if a suspected case showed an epidemiological linkage with a confirmed case—or if the individual reported multiple sexual partners, was positive for anti-MPXV IgM, or was positive on a molecular screening test for orthopoxvirus. A case was confirmed if the individual was positive on a molecular test for MPXV [23]. Skin lesion samples were the foremost clinical samples for diagnosis. 

Data in the present study were collected from 1 June to 1 August 2022. All available clinical samples for each suspected case were tested for MPXV using a real-time PCR assay. Viral DNA was extracted from samples using the Qiagen EZ1robot system (Qiagen, Milano, Italy) and assayed using Real-Star Orthopoxvirus PCR Kits (Altona Diagnostics GmbH). Samples positive on this PCR test were subjected to a second real-time PCR assay specific to MPXV to confirm the infection [24]. The viral strains identified from the first two confirmed cases were subjected to whole-genome sequencing (WGS). MPXV DNA was re-extracted using the Qiagen EZ1robot system (Qiagen). A Nextera XT paired-end library (Illumina, San Diego, CA, USA) was prepared using 1 ng of extracted DNA. The library was sequenced on the MiSeq platform using paired-end sequencing, with a read length of 150 nucleotides (nt) and a mean insert size of 275 nt. For Nanopore sequencing, libraries were prepared from 5 ng of genomic DNA (gDNA) using a rapid PCR barcoding kit (SQK-RPB004; Oxford Nanopore Technologies, Oxford, UK), and were sequenced on a MinION device. A hybrid Nanopore–Illumina de novo assembly was performed using SPAdes. The final genome assembly was completed by manually inspecting and curating the sequences of the large inverted terminal repeats obtained by the de novo hybrid assembly and adding them to the central genome [25]. Phylogenetic analysis was performed using the Nextstrain web app (https://nextstrain.org/, accessed on 17 August 2022). The genome sequences of these viruses were submitted to EpiPox GISAID (https://gisaid.org/, accessed on 17 August 2022) under the accession numbers EPI_ISL_13362760 and EPI_ISL_13362764.

## 3. Results

From 1 June to 1 August 2022, ten cases of Monkeypox were identified in the Apulia region of Italy (Table 1). 

Of these 10 patients, 8 were young adult males, including 6 MSMs, and 2 were female. The average age of all 10 patients was 36.7 years (range: 25–71 years). Only one subject (pt n. 3) was vaccinated against smallpox. Four were also positive for HIV infection and four required hospitalization, but none showed critical symptoms. All patients recovered within a few weeks. Nine subjects reported recent sexual exposure, with three patients (patients 2, 3, and 7) reporting previous sexual contact with an individual suspected of being infected with MPXV. In all 10 patients, the appearance of skin lesions was preceded by a non-specific systemic syndrome, which included fever, shivering and sweating, and lymphadenopathy. 

The first female case was a 71-year-old woman vaccinated for smallpox, with no recent travel history, who reported sexual intercourse with a 56-year-old man with lesions. The man, who was vaccinated for smallpox, was negative for MPXV. At the time of sampling, the skin lesions were healing and the onset of symptoms was reported to have occurred more than three weeks earlier. He reported not having sexual intercourse with another man during the previous weeks and did not consult with any physician for the skin lesions. Unfortunately, serological tests (detection of anti-Monkeypox IgM and IgG) on this probable case were not performed. The second female was a 27-year-old woman who presented with lesions on her face and palms, but none in the genital area. She also showed lymphadenopathy. The only possible risk factors were her participation in a pride party at a local disco and attendance of a concert at a famous holiday venue in the South of Apulia. 

Table 2 shows the results of molecular tests performed on available samples from these patients. The median time from symptom onset to sampling collection was 8 days (IQR: 5.3–10.5 days). At the time of diagnosis, 7 of 10 skin lesion samples had a high viral load of MPXV DNA (quantification cycle value range: 16–21). Six of nine whole blood samples and six of eight nasopharyngeal swab samples also tested positive for MPXV.

**Figure 1:**
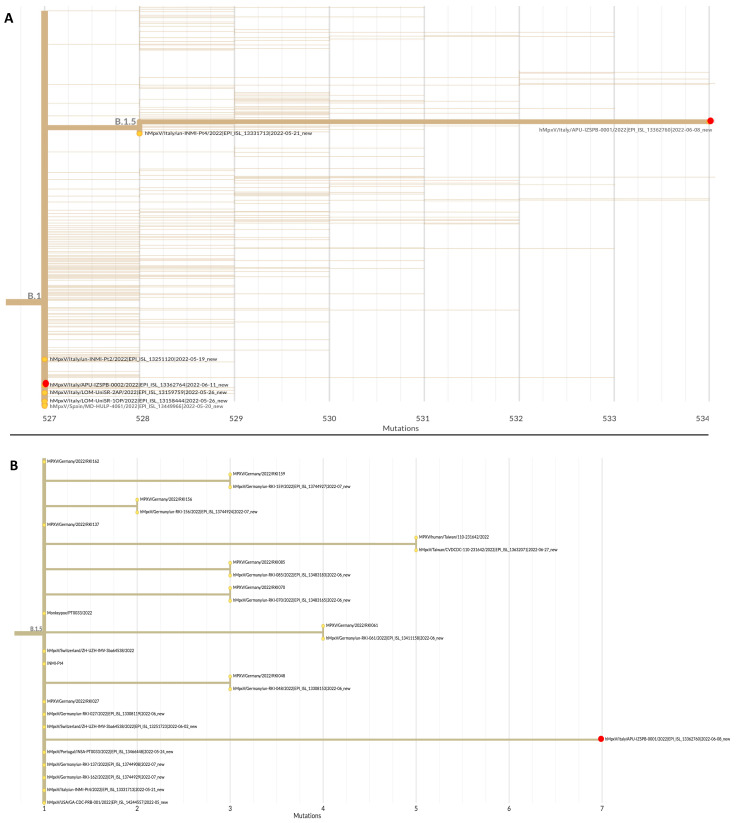
(**A**) Phylogenetic tree of the DNA sequences of samples from patients 1 and 2 in the Apulia region of Italy (red dots), and the sequences of representative Monkeypox genomes downloaded from the GISAID website (https://gisaid.org/, accessed on 17 August 2022); (**B**) phylogenetic tree of the DNA sequences of samples from patient 1 (red dot) and other sequences of representative Monkeypox genomes belonging to the same lineage of B.1.5. Accession numbers, the country of origin, and years are indicated. Phylogenetic analysis was performed using the Nextstrain web app (https://nextstrain.org/, accessed on 17 August 2022).

MPXV DNA samples from patients 1 and 2 were subjected to WGS. Both sequences belonged to Clade 3, lineage B.1.5 and B.1, respectively (Figure 1). A phylogenetic tree showed that the sequence identified in patient 1 was related to sequences from Europe (Germany, Switzerland, Italy, and Portugal) and the USA, whereas the sequence identified in patient 2 was closely related to the sequence of a patient residing in Milan (Italy) and to a sequence from Spain. In fact, patient 2 reported sexual contact with a male subject in Milan.

## 4. Discussion

Many countries are experiencing an upsurge in patients infected with the Monkeypox virus, making the global distribution of infections in the human population a rising concern. Current evidence indicates that MPXV remains zoonotic but is now able of spreading effectively in humans, particularly in highly sexually active networks of unimmunized young men [26]. At the beginning of the 2022 outbreak, gatherings in the Canary Islands of Spain acted as a major amplifying event in Europe [27]. In Italy, the number of cases increased during the last week of July [22]. This recent multinational outbreak of Monkeypox cases has revealed a changing epidemiological trend, with a high proportion of cases occurring among MSM, reaching 87% in Spain [28,29]. Of the eight male patients described in the present study, six (75%) were MSM. At present, however, the proportion of infected patients who are MSM is not yet publicly available in Italy. The expansion of the outbreak within this high-risk community may be linked to this population being too young to have been vaccinated for smallpox as children [30]. The cessation of smallpox vaccination has created an ecological niche in which MPXV can easily spread from human to human [31]. By contrast, patient number 3 in the present study was a 71-year-old woman who had been vaccinated for smallpox as a child [32]. Blood and nasopharyngeal swab samples from this patient, who recovered from MPXV in a few weeks, tested negative for viral DNA only 5 days after symptom onset. These findings suggest that, despite the progressive waning immunity in older subjects, previous smallpox vaccination could confer a cross-immunity, which could produce a mild disease with a favorable outcome following infection with MPXV [33]. Moreover, prior vaccination for smallpox could have accelerated virus clearance. 

At the beginning of August, in Italy, nearly 1% of MPXV-infected individuals (5/545) were female [22]. The identification of a woman (patient 10) without any sexual risk factor could be considered a sentinel event, indicating a possibility of wider risk for community spread of the virus. An MPXV case in a patient in his 20s without any sexual exposure who returned from the United Kingdom to the United States has been recently described [34]. This patient reported his attendance at a large crowded outdoor event, at which he had close contact with other people for a few hours. Moreover, the first case of MPXV in a child without risk factors has been identified in the Netherlands [35]. These findings should raise awareness among clinicians about the possible spread of MPXV in the general population, even without sexual exposure. The atypical manifestation of cases belonging to the ongoing outbreak could lead to a higher proportion of unrecognized infections, increasing the difficulty of breaking the transmission chain [7]. 

Four cases identified in the Apulia region were positive for HIV infection. Although the overall number of cases remains low, findings in HIV-infected patients in Apulia are in agreement with findings reported throughout Europe (36%) [4]. All affected patients in Apulia presented with systemic symptoms, and four required hospitalization. The high proportion of cases involving hospitalization was mostly due to the need for strict isolation. Overall, these patients showed favorable outcomes, including low disease severity and low rates of morbidity and mortality, as reported elsewhere [29].

MPXV DNA was present in almost all the biological samples collected from patients in Apulia. Patients with high viral loads in skin lesions were more prone to positive blood samples. MPXV DNA has been identified in many types of samples other than skin lesions, including saliva, seminal fluid, and rectal swabs [11,17]. Lesion location in the genital or perianal area was significantly associated with the role of the patients regarding sexual practices [29]. The detection of MPXV DNA in seminal fluid suggests that Monkeypox can be regarded as an emerging sexually transmitted infection [29]. Nevertheless, the origin of MPXV infection in a young woman with no sexual risk factors and no lesions in the genital area is unclear.

Whole-genome sequencing showed a close phylogenetic relationship between strains isolated in the present study and other strains belonging to the 2022 MPXV outbreak. In particular, the DNA sequence of the virus from patient 1 was related to sequences from other European countries and the USA, despite this patient reporting no travel or sexual contact with other infected individuals. The viral sequence from patient 2 was closely related to that of an infected patient in Milan, probably via direct sexual contact. The fact that the two sequences belonged to different lineages underlines the possibility of multiple independent introductions of the virus in our region. Because phylogenetic analysis may aid in reconstructing the chain of contagion, genomic surveillance may become a key strategy for controlling the spread of MPXV.

The present study had several limitations. First, probable MPXV could not be confirmed in two cases because serologic data were lacking. Second, DNA sequences from only two patients were subjected to WGS. 

In dealing with MPXV, timely clinical suspicion and a rapid diagnosis are crucial [36]. A major epidemiologic concern is the knowledge gaps regarding MPXV among healthcare workers, which may contribute to the global spread of the pathogen [36,37]. A recent study showed that forming a tailored campaign for healthcare workers is urgently needed [36]. Strengthening surveillance and control activities, together with the vaccination of high-risk households and close contact, are considered the main measures to limit the spread of MPXV in the current outbreak [38]. In Italy, the MVA-BN smallpox vaccine (Imvanex) has been offered as pre-exposure prophylaxis in high-risk individuals—such as laboratory staff members with possible direct exposure to the orthopoxvirus—and gay, transgender, bisexual, and other MSM subjects with specific risk factors [39]. 

## 5. Conclusions

The rapid increase in MPXV cases in non-endemic countries, and the detection of infection in subjects other than MSM, including those without sexual risk factors, raises concerns about the community spread of such a virus. Methods of evaluating the global diffusion of this ongoing outbreak should include genomic surveillance as an essential tool for monitoring and tracking the evolution of this pathogen with a potentially high public health impact. Improving the timely diagnosis and pre-exposure vaccination of high-risk populations may help keep this outbreak under control.

## Figures and Tables

**Table 1:** Clinical and epidemiological characteristics of patients in the Apulia region (Southern Italy) with Monkeypox.

Patient Number	Sex	Age, Year	Smallpox Vaccine	Travel History (Country)	Sexual Behavior	Sexual Exposure (No. of Partners during the Previous 3 Weeks)	HIV Status	Systemic Symptoms	Location of Skin Lesions	Hospitalization
**1**	Male	30s	No	No	Heterosexual intercourse	Yes (4)	Negative	Yes	Oral, genital area	No
**2**	Male	30s	No	Yes (Italy)	MSM ^1^	Yes (2)	Positive	Yes	Face, arms, legs, trunk, back, genital area	No
**3**	Female	70s	Yes	No	Heterosexual intercourse	Yes (2)	Negative	Yes	Leg, arms, trunk, back, genital area	Yes
**4**	Male	30s	No	Yes (Italy)	MSM	Yes (2)	Negative	Yes	Face, arm, trunk, back	No
**5**	Male	30s	No	Yes (Germany, Italy)	MSM	Yes (>10)	Positive	Yes	Face, palms, oral, arms, genital area	No
**6**	Male	20s	No	Yes (Egypt, France)	Heterosexual intercourse	Yes (2)	Positive	Yes	Face, palms, soles, arms, trunk, genital area	Yes
**7**	Male	30s	No	Yes (Italy)	MSM	Yes (1)	Negative	Yes	Face	No
**8**	Male	30s	No	Yes (Spain)	MSM	Yes (5)	Negative	Yes	Genital area	Yes
**9**	Male	30s	No	Yes (Italy)	MSM	Yes (5)	Positive	Yes	Genital area	No
**10**	Female	20s	No	Yes (Italy)	No sexual intercourse	No	Negative	Yes	Face, palms	Yes

^1^ MSM = men who have sex with men.

**Table 2:** Characteristics of clinical samples of patients in the Apulia region (Southern Italy) with Monkeypox.

Patient	Date of Sample Collection	Days from Symptom Onset	Sample	Results	Cq (Quantification Cycle)
1	8 June 2022	9	Skin lesion swab	Positive	16
	8 June 2022		Blood	Positive	35
	8 June 2022		Nasopharyngeal swab	Positive	30
2	11 June 2022	11	Skin lesion swab	Positive	17
	11 June 2022		Blood	Positive	32
	11 June 2022		Nasopharyngeal swab	Positive	30
3	20 June 2022	5	Skin lesion swab	Positive	35
	20 June 2022		Blood	Negative	
	20 June 2022		Nasopharyngeal swab	Negative	
4	21 June 2022	7	Skin lesion swab	Positive	20
	21 June 2022		Blood	Positive	35
	21 June 2022		Nasopharyngeal swab	NA ^1^	
5	22 June 2022	4	Skin lesion swab	Positive	20
	22 June 2022		Blood	Positive	32
	22 June 2022		Nasopharyngeal swab	Positive	30
6	23 June 2022	6	Skin lesion swabs	Positive	17
	23 June 2022		Blood	Negative	
	23 June 2022		Nasopharyngeal swab	Positive	31
7	25 June 2022	12	Skin lesion swab	Positive	23
	25 June 2022		Blood	Positive	35
	25 June 2022		Nasopharyngeal swab	Negative	
8	14 July 2022	2	Skin lesion swab	Positive	20
	14 July 2022		Blood	NA	
	14 July 2022		Nasopharyngeal swab	NA	
9	27 July 2022	17	Skin lesion swab	Positive	21
	27 July 2022		Blood	Negative	
	27 July 2022		Nasopharyngeal swab	Positive	40
10	2 August 2022	8	Skin lesion swab	Positive	23
	2 August 2022		Blood	Positive	33
	2 August 2022		Nasopharyngeal swab	Positive	32

^1^ NA = not available.

## Data Availability

Data are available on request from the corresponding author.
